# Performance in delayed non-matching to sample task predicts the diagnosis of obsessive–compulsive disorder

**DOI:** 10.1038/s41398-019-0667-3

**Published:** 2019-12-10

**Authors:** Redwan Maatoug, Benoît Le Goff, Jean-Yves Rotge, Pr Nemat Jaafari, Pr Olivier Guillin, Pr Bruno Millet

**Affiliations:** 1 Departement de Psychiatrie adulte, boulevard de l’Hopital, Hopital Universitaire de la Pitie Salpetriere, Assistance Publique - Hopitaux de Paris, 75013 Paris, France; 2 Service Hospitalo-universitaire, CH du Rouvray, 4 rue Paul Eluard, 76300 Sotteville-les-Rouen; unite Inserm U1079 Faculte de medecine et de pharmacie, 76000 Rouen, France; 3 CIC INSERM U802, CHU de Poitiers, Unite de recherche clinique intersectorielle en psychiatrie du Centre Hospitalier Henri Laborit, Poitiers, 86022 France

**Keywords:** Psychiatric disorders, Diagnostic markers

## Abstract

Electrical stimulation studies have recently evidenced the involvement of orbitofrontal cortex (OFC) in obsessive–compulsive disorder (OCD). In addition, lateral OFC is activated in healthy subjects during delayed non-matching-to-sample task (DNMS). In the present study, we hypothesized that OCD results from a specific defect of lateral OFC processing that can be evidenced via a DNMS task. To this end, we compared the DNMS performances of 20 OCD patients vs 20 demographically matched healthy controls. As predicted, our results showed that OCD patients performed worse than healthy controls at DNMS task. To test for the specificity of this behavioral impairment, we furthermore compared OCD patients and healthy subjects on a different task not involving directly the lateral OFC: the delayed match-to-sample task (DMS). As expected, OCD patients are more impaired for both the DNMS and the DMS task, compared with healthy subjects. Moreover, OCD patients tend statistically to perform worse for the DNMS task than for DMS task. Our results suggest the DNMS task specifically target the malfunctioning areas in OCD, such as the lateral OFC. In light of these results, lateral OFC should therefore be the focus of future therapeutic interventions.

## Introduction

Obsessive–compulsive disorder (OCD) is a leading cause of disability worldwide^1^. Many epidemiologic studies have rated the lifetime prevalence of OCD ranging between 1.9 and 3.0%^2^. It is a chronic neuropsychiatric disorder marked by intrusive and disturbing thoughts, images or impulses (obsessions) and repetitive, ritualized behaviors (compulsions) that the patient feels driven to perform. Most of the OCD patients complain, first from checking symptoms (feeling compelled to check the doors are locked—the gas is off), second from washing symptoms (cleaning, hand-washing compulsions, and contamination) and finally from other symptoms such as the taboo thoughts or the need for symmetry^3^. Over time, compulsions can become so time consuming (> 1 h/day) or onerous that they engender anxiety themselves. These symptoms produce profound distress and most frequently interfere heavily with daily functioning.

Approximately 70% of patients experience significant symptomatic relief with appropriate pharmacotherapy^4^. Selective serotonin reuptake inhibitors (SSRIs) are the main pharmacological treatment. The combination of medication with cognitive and behavioral therapies (CBT) is often used. However, treating patients suffering from OCD disorders remains a critical clinical challenge.

Today, preclinical literature strongly suggests that OCD can be characterized by abnormalities in fronto-striatal circuits involved in learning and habitual control^5^. The focus on such abnormalities has led practitioners to develop links with neurosurgeons to use high-frequency deep brain stimulation (DBS) for the most severe treatment-resistant OCD patients, as well as non-invasive techniques such as repetitive transcranial magnetic stimulation (rTMS). Nowadays, the improvement of these stimulation procedures allows to stimulate deeper thanks to the development of new specific coils and more accurately thanks to neuronavigation device. That is why, the more accurate we manage to be in determining the brain regions involved in OCD, the better we would be able to treat the patients’ resistant to pharmacotherapy.

In order to specify a neuroanatomical target of non-invasive therapeutic stimulations and neurosurgery within the fronto-striatal circuits, research has suggested that a focus on the orbitofrontal cortex (OFC), which belongs to the prefrontal cortex (PFC) and occupies the central part of the frontal lobe, may be highly relevant. Baxter et al.^6^, using neuroimaging to compare a group of patients suffering from OCD with a sample of healthy controls (HC), have first demonstrated a bilateral hyperactivity of the OFC in patients compared with controls. Since then, several functional neuroimaging have replicated these findings, reporting either left-sided, right-sided, or bilateral PFC hyperactivation or hypermetabolism in OCD^7^. Finally, Rotge et al.^8^ showed that altered left lateral OFC activity may not only be a crucial marker of OCD symptomatology but could also play a role in its genesis.

Researchers have also been looking for a biomarker able to reflect the response to a therapeutic intervention for OCD. Concordantly to the previously described research works on the implication of OFC in OCD, studies have shown a correlation between a decrease in OFC hypermetabolism in OCD patients with an effective treatment. First Rubin et al.^9^ and then Nakao et al^10^. have confirmed that pharmacological treatments used for OCD patients (CBT and SSRIs) lead to a decrease in OFC hypermetabolism. Moreover, Nuttin et al.^11^ have proved that treatment of OCD using brain simulation techniques leads to a decrease in OFC hypermetabolism. Next, Le Jeune et al.^12^, in treatment-resistant OCD patients, have established a correlation between a decrease in scores on the Yale-Brown obsessive–compulsive scale (YBOCS) and a decrease in PFC metabolism during subthalamic nucleus (STN) stimulation, knowing that a small score at the YBOCS is a synonym of remission for OCD patients. Then, Nauczyciel et al.^13^ have demonstrated that the OFC might be reached by a non-invasive brain stimulation such as rTMS and that stimulating this region may benefit patients suffering from OCD. Considered together, these results strongly suggest that altered OFC activity is a functional biomarker of OCD and a potential predictor of response to treatment.

**Fig. 1:**
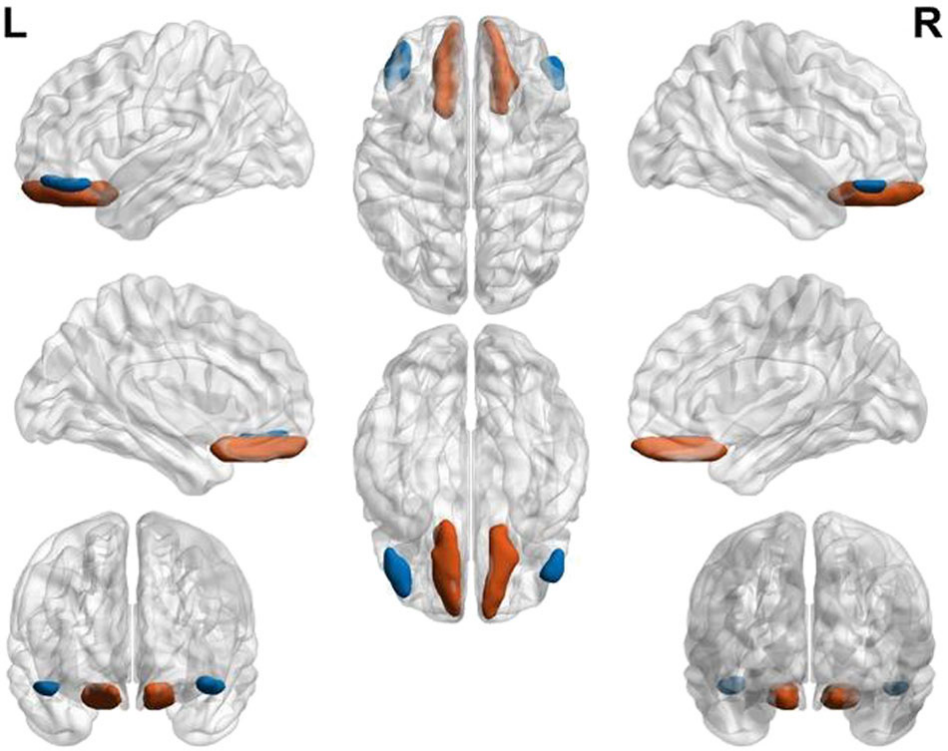
Location of the OFC region. Red = the mOFC; blue = the lOFC.

Regarding its function, the OFC has been widely implicated in reinforcement processing, both in animals and humans, which appeared to be deficient in patients suffering from OCD. It is also clear that there is a functional heterogeneity within this region and recent fMRI studies in humans have suggested distinct roles in reinforcement processing for different subregions of the OFC^14–17^. As revealed by a large meta-analysis, one of the most reliable distinctions made is the trend for medial regions of the OFC (mOFC) to be more associated with positive reinforcement, whereas lateral regions (lOFC) seem to be more often associated with negative reinforcement, which is characteristics of OCD (Fig. 1). Moreover, it has been hypothesized that the results obtained in lesion studies may reflect a critical role of the lOFC in inhibiting previously established responses which cease to be appropriate^18,19^.

The distinction between lOFC and mOFC dysfunction in OCD was suggested by an early positron-emission tomography study reporting that OCD symptoms were positively correlated with the metabolism in the anterolateral OFC and were negatively correlated with the metabolism in the posteromedial OFC regions^20^.

Subsequent fMRI studies reported positive correlations between hyperactivation of the lOFC and OCD symptoms severity during the performance of the serial reaction time task^21^ and during symptoms provocation^22,23^. Studies of OCD treatment response have shown that lOFC hyperactivity prior to therapy predicts the subsequent response to serotonergic reuptake inhibitors; the less the magnitude of OFC hyperactivity, the better the response to treatment^24^. In 2008, Chamberlain et al.^25^ demonstrated that impaired probabilistic reversal learning in OCD is associated with hypofunction of bilateral lOFC, both in patients with OCD and in their unaffected relatives, suggesting that this abnormality could even represent an endophenotype of this disorder. Similarly, Remijnse et al.^26^ showed decreased lOFC activation in OCD during probabilistic reversal learning. In 2013, Burguière et al.^5^ have also reinforced this idea by successfully suppressing compulsions in a mouse model after optogenetic stimulation of lateral orbitofronto–striatal pathway.

Based on this literature review, our hypothesis is that the lOFC is involved in OCD and can be a relevant target to treat this disorder using non-invasive (rTMS) and invasive (deep brain stimulation) techniques. We believe that both the treatments of OCD and the monitoring of the efficiency of these treatments, especially DBS, rTMS, and the pharmacological ones, can be improved using a task that would be performed differently by OCD patients and HC and targeting a superficial brain area such as the lOFC.

This behavioral task should present the following characteristics: simple and short, with results easy to assess and involving the lOFC.

No study has found any difference between OCD patients and HC in terms of correct answers to a specific cognitive task. Millet et al.^27^ in 2013 have compared OCD patients and HC using several neuropsychological tests such as Hopkins test, word fluency, rey complex figure copy, trail making test, tower of London, Stroop test, and object alternation task. They only found statistical difference between OCD patients and HC in terms of response time. They did not find any difference concerning the number of correct answers.

In a study of Elliott D.F. et al.^18^, which demonstrated that the lOFC is more activated in the delayed non-matching-to-sample (DNMS) task than in the delayed matching to sample (DMS) task, we decided to focus on the DNMS. To our knowledge, the DNMS task has never been tested in OCD patients. However,only two studies have already studied the DMS task in OCD patients vs HC without showing any significant statistical difference in terms of correct answers^28,29^.

In this study, our primary objective is first to compare performances of OCD patients vs HC on the DNMS task, second on the DMS task, and last compare the performances of OCD patients on the DNMS task vs DMS task in terms of correct answers and reaction time. Our hypothesis is that OCD patients are more impaired in the DNMS task than in the DMS task.

## Methods

### Participants

Patients with OCD, from 18 to 65 years old, were recruited prospectively over 4 months from the adult psychiatry department of La Pitié Salpêtrière Hospital, in Paris (France). The diagnosis of OCD has been made by senior psychiatrists according to the DSM-5 (American Psychiatric Association, 2013). All patients were considered OCD treatment-resistant because they did not respond to at least two different SSRIs and to a CBT. Axis-1 screening was performed with the Mini-International Neuropsychiatric Interview (MINI), symptoms and severity of OCD were measured using the YBOCS and the depressive symptoms were assessed with a Hamilton depressive rate scale (HDRS). The exclusion criteria were: psychotic spectrum disorders, bipolar disorder, severe depression (HDRS > 18). Comorbid mild and moderate depression as well as anxious disorders are common in OCD patients, and we chose not to exclude those patients. For each patient, the age of onset, the time lapse before beginning an antidepressant or a psychotherapy, the pharmacological treatment history and the number of rTMS sessions received before the assessment were gathered.

HC were recruited from the general population. They were screened by a senior psychiatrist to exclude current or past psychiatric disorders using the MINI and the HDRS. Controls were matched with OCD patients for age (±5 years), gender, and years of education.

This study was conducted in accordance with the Code of Ethics of the World Medical Association (Declaration of Helsinki) for experiments involving humans and was approved by the Committee for the protection of patients (“Comité de Protection des Personnes”: CPP) of La Pitié Salpêtrière Hospital on March 2016. All patients signed a written consent to participate in this study after they have been given a complete description of the study.

### Instruments

The patients performed the DNMS task and the DMS task using the PEBL software^30^. In order to counteract an eventual order effect, the sequence of the two tests was systematically alternatively changed. Consequently, half of the subjects performed the tests in the following order: DNMS/DMS and the other half in the reverse order DMS/DNMS.

**Fig. 2:**
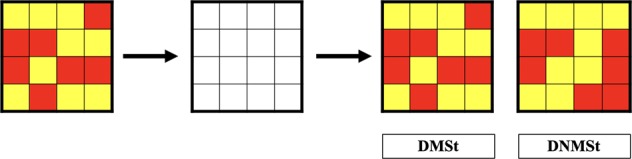
Assesment of the working memory in healthy subjects vs OCD patients. Delayed matching to sample and delayed non-matching to sample tasks.

As far as the DNMS and DMS tests are concerned, we used the methodology of Elliott et al.^18^ that studied OFC activity in healthy subjects performing the DNMS and the DMS tasks. In both tasks, subjects were initially shown during 1 s a matrix filled with 16 red and yellow squares characterized as a complex and abstract visual stimulus. Then after a delay of 5 s, they were presented with two different matrices, one of which was the sample stimulus, identical to the first matrix presented. In the DNMS task, the subject was asked to choose the novel stimulus, whereas in the DMS the subject was asked to choose the familiar stimulus (Fig. 2). The subject had no time limit for choosing the correct stimulus related to the task. This task was repeated 30 times for every subject. For each subject, we have measured two main parameters; the number of correct answers and the response time during each trial. The subject had no time limit to answer questions. Before starting the recorded session, a test of five trials was done for every subject to check the understanding to the task

### Statistical analysis

First, we measured parametric correlation (Pearson test) on sociodemographic data to compare continuous variables between OCD patients and HC. Then, Student’s *t* tests were performed to compare the results to the DNMS and the DMS tasks between OCD patients and HC using two parameters: the number of correct answers and the response time. Subsequently, in order to find out the most specific task for OCD patients, we determined that task among DNMS and DMS appeared to be the most difficult for OCD patients. Finally, a bootstrapping method was used to confirm the previous results from the Student’s *t* test. The bootstrapping method relies on random sampling with replacement. It is an appropriate way to control and check the robustness of the results on a sample size with <40 subjects^31^.

We have performed the statistical analysis tests using RStudio software in its 3.5.0 version.

The threshold for statistical significance was set at *p* < 0.05.

## Results

A total of 42 subjects entered the study. The final sample for analysis consisted of 40 subjects (95.24%) as one patient, severely depressed, was excluded (HDRS = 22) and a HC, treated by psychotropic medication for anxious and depressive symptoms, was also excluded. We included 20 OCD patients (mean age = 41 ± 8.9 years old; range = 27–59 years; mean education = 14 ± 2.5 years; mean age at onset = 17 ± 8.9 years old; mean YBOCS score = 29 ± 5.3) and 20 HC (mean age = 40 ± 9.1 years old; range = 29–56 years; mean education = 15 ± 3.8 years). Table 1 shows the demographic characteristics of both groups and the results of the correlation tests comparing OCD patients and HC. The OCD sample did not differ significantly from the control sample with respect to age (*p* = 0.4) and education (*p* = 0.4) as we can see in the Table 1.

**Table 1:** Group mean (SD) sociodemographic and clinical variables for OCD patients and healthy controls (HC).

	OCD	Controls	*p*
Number of subjects	20	20	
Gender (m;f)	11; 10	11; 10	
Age (years)	41 (8.9)	40 (9.1)	0.4
Education (years)	14 (2.5)	15 (3.8)	0.4
HDRS	6 (4.8)	0 (0.8)	
YBOCS	29 (5.3)		
YBOCS obsessions	14 (3.2)		
YBOCS compulsions	15 (2.6)		
Age at onset (years)	17 (8.9)		
Duration of illness without treatment (years)	9 (10.5)		
Number of rTMS (sessions)	17 (17.1)		

In the OCD group, 13 patients had no comorbidities and only five of them had depressive symptoms at the HDRS. Most of the OCD patients have presented checking symptoms (feeling compelled to check—such as checking doors are locked or the gas is off) as the first dimension and washing symptoms (cleaning or hand-washing compulsions) as the second dimension, which is concordant with the literature cited in the introduction section. Except two patients, all the others have taken at least one antidepressant. In addition to an antidepressant, four patients have taken a benzodiazepine and seven have taken antipsychotic at the moment of assessment (Table 2).

**Table 2:** Numbers of patients in the OCD group with lifetime comorbidities, OCD symptom dimensions, and medication at inclusion.

Comorbidities		Dimension		Medication	
No comorbidities	13	Checker	11	No medication	2
Mild depression	3	Washer	6	At least one antidepressant	18
Moderate depression	2	Other	3	Clomipramine	12
Panic disorder	2			Antipsychotic	7
Social phobia	3			Benzodiazepine	4
PTSD(post-traumatic stress disorder)	1				

For each patient, only the main dimension is represented in the table

The comparative analysis of performance in the DNMS the DMS tasks between OCD patients and HC using a Student's *t* test has proven to be statistically significant. As expected, OCD patients perform worse than HC for the DNMS task *(t* = −2.687, *p* = 0.010***) and for the DMS task *(t* = −2.089, *p* = 0.043*) in terms of correct answers. Otherwise we can observe that this difference between OCD patients and HC seems statistically stronger for the DNMS task than for the DMS task *(*Cohen’s *d* 0.85 vs 0.66). Furthermore, the response time for OCD patients is greater than the response time for HC performing the DNMS task (*t* = 2.519, *p* = 0.016***) and the DMS task (*t* = 2.332, *p* = 0.025**). Regarding the OCD patients only, there is a trend showing that they perform worse for the DNMS task than for the DMS task (Table 3).

**Table 3:** Comparison of performances in DMS and DNMS between OCD patients and healthy controls using Student’s *t* test.

	Mean (OCD; HC)	*t*	*p*	Cohen’s *d*
Correct answers in OCD patients vs HC for the DNMS	26.00; 28.35	−2.687	0.010***	0.85
Response time in OCD patients vs HC for the DNMS	2971; 2125	2.519	0.016***	0.80
Correct answers in OCD patients vs HC for the DMS	26.30; 27.65	−2.089	0.043*	0.66
Response time in OCD patients vs HC for the DMS	2623; 1889	2.332	0.025**	0.74
Correct answers DNMS vs DMS for OCD patients	26.00; 26.30	−0.307	0.082	0.62
Response time DNMS vs DMS for OCD patients	2971; 2623	2.285	0.03*	0.24
Correct answers DNMS vs DMS for HC	28.35; 27.65	1.788	0.09	0.33
Response time DNMS vs DMS for HC	2124; 1889	1.423	0.170	0.46

*t* *t* test; *p* *p* value; *df* degrees of freedom; *significance; response time in ms calculated only for correct answers

We then decided to confirm this trend using a bootstrapping method with 100 permutations and replacement. The results for the bootstrap (mean DNMS task correct answers = 25.881, mean DMS task correct answers = 26.324, *t* = −4.593, *df* = 19) show that the trend becomes significant with a larger sample.

Finally, a multivariate linear regression was calculated to predict the OCD severity at the YBOCS based on the reaction time and the correct answers at the DNMS task. We found a negative correlation between the OCD severity at the YBOCS and the correct answers at the DNMS task (*β* = −1.56; *p* = 0.027*; SE = 0.65). No correlation was found between the OCD severity at the YBOCS and the reaction time at the DNMS task.

Our results are consistent with our hypothesis saying that OCD patients would be more impaired performing the DNMS task that the DMS task. We have also shown that the more severe a patient is at the YBOCS the less he will perform at the DNMS task.

## Discussion

In this study, we compared the performances of OCD patients vs HC performing two neuropsychological tasks: the DNMS and DMS tasks. Our focus laid mainly on the performances for the DNMS task. This task was chosen for two main reasons; 1. because previous studies, such as the one of Elliott et al.^18^, have demonstrated that performing this task involves the lOFC; and 2. because a dysfunction of the lOFC is associated with OCD. Our goal in this study was then twofold; first bringing some new evidence that lOFC activity is a biomarker of OCD and second, highlighting the DNMS could be a relevant task for studying OCD patients and monitoring the efficiency of treatments, particularly the ones involving non-invasive and invasive brain stimulation and pharmacological treatments, as they directly impact the OFC. First of all, our results confirm that OCD patients perform statistically worse than HC for the DNMS and the DMS tasks considering the number of correct answers and the response time. Then, using a *t* test confirmed with a bootstrapping, we proved that OCD patients seem to be more impaired for the DNMS task than for the DMS task. Finally, we have also shown that the more severe a patient is at the YBOCS the less he will perform at the DNMS task.

Contrary to the results of Martoni et al. and Ciesielski et al.^28,29^, our study states that OCD patients perform worse than HC for DMS task. However, this difference may be explained by a different methodology; first Ciesielski et al. followed four OCD patients only and, although Martoni et al. used a sample of 42 OCD patients, they chose not to monitor the response time. We then demonstrated that OCD patients take significantly more time to perform the tasks but they not only take significantly more time than HC, they also make more mistakes.

The two tasks tested, non-matching (DNMS) and matching (DMS) involve short-term memory processes and the execution of a choice response. In a previous neuroimaging study from Elliot et al.^18^ the medial activation associated with the matching condition was explained in terms of a need to monitor the familiarity and associated reward value of stimuli. The lateral activation associated with non-matching, however, could be explained in terms of a crucial difference between the tasks at the choice stage. In adult humans, unlike children and animals, matching is a more natural process than non-matching;^32^ the instinctive response at the choice stage is to the familiar rather than the novel stimulus. Thus, making the choice in non-matching involves inhibiting an instinctively preferred response in order to make a correct one. Inhibition can be seen as a particular feature of making choices, especially in circumstances where an established or salient response may not always be appropriate. Such a role for lOFC is consistent with previous studies implicating the OFC in response inhibition tasks, such as the classic go-no-go tasks^33–35^. Recently, the role of lOFC in response inhibition has been suggested in few animal studies examining neural correlates at the single-unit level^36^. Considering the results of our study, we can conclude that OCD patients are characterized by a lack of inhibition, resulting from a dysfunction of the lOFC and leading them to have poorer performances in making the choice in non-matching comparing with healthy subjects. Our study then establishes DNMS as a relevant task to monitor the efficiency of OCD treatments.

However, other studies would be useful to support that these findings can be replicated or to assess whether our results are explained by the take of a psychotropic treatment in the OCD group. Indeed, one of the limitations of our study is that some of our patients were undergoing pharmacological treatment at the time of study. This is more representative of the general OCD population, but permits a possible confound when comparing with HC who are receiving no psychotropic medication, although treatment in our sample was heavily weighted toward SSRIs, found in the literature not to affect neurocognitive function^37^. It could also be very interesting to replicate this study with a larger number of participants and a four- choices version of DNMS test, such as the one used in Martoni et al.’s study.
