# Correction: Performance in delayed non-matching to sample task predicts the diagnosis of obsessive–compulsive disorder

**DOI:** 10.1038/s41398-020-0735-8

**Published:** 2020-01-29

**Authors:** Redwan Maatoug, Benoît Le Goff, Jean-Yves Rotge, Nemat Jaafari, Olivier Guillin, Bruno Millet

**Affiliations:** 1 Departement de Psychiatrie adulte, boulevard de l’Hopital, Hopital Universitaire de la Pitie Salpetriere, Assistance Publique - Hopitaux de Paris, 75013 Paris, France; 2 Service Hospitalo-universitaire, CH du Rouvray, 4 rue Paul Eluard, 76300 Sotteville-les-Rouen; unite Inserm U1079 Faculte de medecine et de pharmacie, 76000 Rouen, France; 3 CIC INSERM U802, CHU de Poitiers, Unite de recherche clinique intersectorielle en psychiatrie du Centre Hospitalier Henri Laborit, Poitiers, 86022 France

**Keywords:** Psychiatric disorders, Diagnostic markers

**Correction to: Translational Psychiatry**

10.1038/s41398-019-0667-3 published online 10 December 2019

The original Article contained a few errors in the author list. The given list was formally: “Maatoug R, Goff BL, Rotge JY, Jaafari PN, Guillin PO, Millet PB”, when it should be “Maatoug R, Le Goff B, Rotge JY, Jaafari N, Guillin O, Millet B.” This has been corrected in the XML, PDF and HTML versions of this Article. The publishers would like to apologise for this error.

